# Hydrophobic carboxymethyl cellulose as a clean-up sorbent in the determination of nitrofuran metabolites in animal-fat samples

**DOI:** 10.1039/d3ra07021b

**Published:** 2023-11-10

**Authors:** Omar A. Thabet, Faisal S. Al Muzini, Abdulaziz M. Atiya, Khalid A. Alamry, Mahmoud A. Hussein, Richard Hoogenboom

**Affiliations:** a Department of Chemistry, Faculty of Science, King Abdulaziz University Jeddah 21589 Saudi Arabia kaalamri@kau.edu.sa mahussein74@yahoo.com maabdo@kau.edu.sa; b Saudi Food and Drug Authority Jeddah 22311 Saudi Arabia; c Chemistry Department, Faculty of Science, Assiut University Assiut 71516 Egypt; d Supramolecular Chemistry Group, Department of Organic and Macromolecular Chemistry, Centre of Macromolecular Chemistry (CMaC)Ghent University Krijgslaan 281 S4 9000 Ghent Belgium

## Abstract

Hydrophobic carboxymethyl cellulose (CMC) biopolymers were fabricated for the removal of fat from food sample matrices. The hydrophobic CMCs were synthesised *via* the esterification of CMC with three alcohols with carbon chains of different lengths, methanol, butanol, and octadecanol, in the presence of sulfuric acid. The structure of the three synthesised hydrophobic CMCs was verified using FT-IR, and the physicochemical properties were investigated by TGA, SEM, and X-ray. Characterization confirmed the successful synthesis of the hydrophobic CMCs and that the hydrophobic groups are embedded in the sorbent biopolymer to interact with fat and reduce the fat content of the sample extract. Moreover, the performance of the fabricated hydrophobic CMCs was studied in two applications: fat removal and the determination of nitrofuran (NF) metabolites in fat samples. In the first application, excellent results were observed for fat removal; the highest percentage of fat removed from food sample extracts was 94.2% and the lowest was 88.5%. Successful results were also observed in the determination of NF metabolites in fat samples, as the final extract was clear and pure using the hydrophobic CMCs, while it was turbid for the control sample. In addition, the recovery of four NF metabolites was in the range of 97–117%. In general, the hydrophobic CMCs showed promising and satisfactory results, with CMC-C18 exhibiting the best results. The NF detection method was validated using CMC-C18 in three spiking levels; 0.5, 1.0 and 1.5 μg kg^−1^. The average recoveries of NF range between 83.3 to 104.3%, and the intra-day precision was determined by coefficient of variation, which was below 10% for all NF. The limit of detection and limit of quantification were between 0.6 to 0.9 and 0.20 to 0.28 μg kg^−1^ respectively. For linearity, the correlation coefficient (*r*^2^) was higher than 0.99 for NF metabolites. Overall, the hydrophobic CMCs can be further developed and safely used as green sorbents in food analysis applications.

## Abstract 2

Hydrophobic-CMC biopolymers were fabricated for the removal of fat from food sample matrices. The CMCs were synthesized *via* the esterification of CMC with three alcohols with carbon chains of different lengths, in the presence of sulfuric acid.

## Graphical abstract

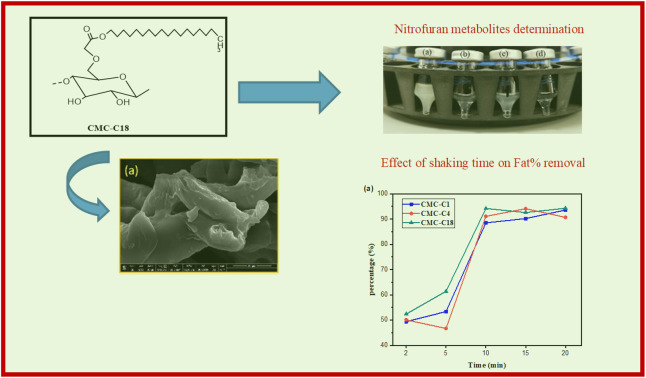

## Introduction

The polysaccharide biopolymer cellulose is an important bioresource with unique properties. For this reason, it has been of research interest over the last few decades. It is the most abundant of the known biopolymers, is easy to obtain, low in cost, safe for humans and the environment, biodegradable, biocompatible, renewable, simple to chemically modify, and has numerous derivatives.^1^ These features make cellulose an ideal material, and as such it has been widely used in various fields, such as in food, medicine, environmental treatment, and the textile industry.^2^ Cellulose is composed of anhydro-d-glucopyranose units (AGUs), which feature three hydroxyl groups that increase the reactivity of the compound.^3^ Therefore, based on this structure, further modification and chemical functionalisation of cellulose have been successfully achieved to generate materials with desired properties, such as cellulose acetate, cellulose nitrate, methylcellulose, and carboxymethyl cellulose.^4^

Carboxymethyl cellulose (CMC) is a well-known anionic derivative of the cellulose family that exhibits high solubility in water and a higher molecular weight than cellulose.^5^ It can be produced by replacing one of the hydroxyl groups in the AGU with a sodium carboxymethyl group. This reaction is achieved *via* alkalization followed by carboxymethylation. In alkalization, the hydroxyl group is activated using reaction media and sodium hydroxide, and after that carboxymethylation is achieved using monochloroacetate salt.^6^ CMC has been widely used in manufacturing and product development due to its unique chemical and physical properties. More than half a million tonnes of CMC are produced annually to be used in various applications, such as in pharmaceuticals, medicine, and food.^7^ In food, CMC is used as a thickener, stabilizer, and emulsifier^8^ as, like cellulose, it is safe, non-toxic, biocompatible, and biodegradable.^9^ Moreover, CMC can be chemically modified according to requirements. Modification can be achieved at the C_2_ and C_3_ hydroxyl groups in the AGU or at the C_6_ carboxyl group.^10^ As a result, the modified CMC can be tuned to have hydrophilic or hydrophobic properties.^10–12^

Nitrofuran (NF) metabolites analysis is one of challenging food analysis applications. They are a group of synthetic antibiotics used in aquaculture, honeybees, and livestock to treat bacterial infection.^13^ The chemical structure of NF features a nitro group embedded in a furan ring, which endows the compound with its bactericidal activity.^14^ There are four well-known compounds in the NF class of antibiotics that are commonly used in animal treatment: nitrofurazone (NFZ), furaltadone (FTD), furazolidone (FZD), and nitrofurantoin (NFT).^15^ These drugs exhibit a short half-life in organisms, and are converted to the metabolites semicarbazide (SEM), 3-amino-5-(4-morpholinyl-methyl)-1,3-oxazolidin-2-one (AMOZ), 3-amino-1,3-oxazolidin-2-one (AOZ), and 1-amino-hydantoin (AHD) after digestion.^16^ Several research studies have addressed the hazards associated with these NF metabolites, as they are considered to be highly toxic carcinogenic and mutagenic compounds.^17^ Therefore, most countries have banned NF usage, and set a value of 1 μg kg^−1^ as a minimum required performance limit (MRPL) for NF metabolites.^18^ In spite of this strict legislation, NFs are still used for treatment purposes in livestock.

To ensure the safety of animal products and monitor the abuse of NF drugs usage, many analytical methods have been developed, such as high-performance liquid chromatography coupled with tandem mass spectrometry (LC-MS/MS). As NF metabolites are low in molecular weight, the majority of analytical methods require the use of derivatisation techniques involving 2-nitrobenzaldehyde to detect NF metabolites reliably by LC-MS/MS.^19^ The most critical step in NF analysis is the clean-up process that has a significant effect on the recovery of the target compounds as well as the purity of the final extractor which is injected in the LC-MS/MS system. The efficiency of clean-up step is important to overcome animal matrix interferences, especially fats interference, which have a significant effect on NF metabolite determination at the MRPL level. Several approaches have been reported for clean-up techniques, for instance, solid phase extraction (SPE),^14,20–22^ liquid–liquid extraction (LLE)^23–26^ and freezing fats filtration.^27,28^ In spite of the diversity of such techniques, they have many limitations such as time consuming, high cost, large amount of used chemicals and low efficiency. Therefore, challenges still exist in terms of fat matrix interferences and the development of a green, low-cost, simple, and accurate clean-up technique is required.

In this study, a fat sorbent is developed using a biopolymer to remove fat interferences in clean-up step for NF metabolites detection analysis in animal-fat samples. The unique derivatives of CMC biopolymer were synthesized and chosen as a sorbent after doing some modification by embedding hydrophobic molecules to absorb fat present in the animal-fat sample. Practically, CMC was reacted with non-polar hydrocarbon chains by esterification *via* an eco-friendly ultrasound-assisted procedure to obtain hydrophobic CMCs. After that, the structures of the synthesized hydrophobic CMCs were verified using Fourier-transform infrared (FT-IR) spectroscopy, thermogravimetric analysis (TGA), scanning electron microscopy (SEM), and X-ray diffractometry (XRD). Finally, the performance of the hydrophobic CMCs was investigated for two applications: fat removal and the determination of NF metabolite residues from fat samples using LC-MS/MS.

## Experimental

### Materials and equipment

All solvents and chemicals were of analytical grade. Carboxymethyl cellulose CMC (Sigma Aldrich), methanol (99.8%, Fisher), 1-butanol (99.8%, Fisher), 1-octadecanol (97%, Alfa Aesar), sulfuric acid (96%, PanReac), ethanol (99.7%, VWR), acetone (99.5%, BDH), ethyl acetate (99.95%, HPLC grade, Fisher), dimethyl sulfoxide (99.5%, Fluka), hydrochloric acid (36%, ROMIL), deionised (DI) water (VWR), 2-nitrobenzaldehyde (2-NP) (Sigma Aldrich), anhydrous ammonium acetate (97%, VWR), anhydrous di-potassium hydrogen orthophosphate (99.0%, Sigma Aldrich), and sodium hydroxide (97%, Merck) were used in the experiments. AOZ, AHD, AMOZ, SEM, AMOZ-d5, AOZ-d4, AHD-13C3, and SEM-(13C,15N_2_) were purchased from Sigma Aldrich.

The equipment used in the polymer synthesis, such as the ultrasonic water bath (Elmasonic-S 60H), 13 000 rpm centrifuge (HERMLE), nitrogen evaporation water bath (Caliper, TurboVap LV), rotary shaker (Heidolph), ESI-LC MS/MS Triple Quad (6500+, sciex), were used for the NF metabolite determination. In regard to characterization techniques, FT-IR spectroscopy (Thermo Scientific, Nicolet iS50 FT-IR spectrometer) was used to identify functional groups, XRD (Bruker Co, D8 Discover, Cu target, 40 kV, 40 mA, wavelength 1.54 Å) was used to study crystallographic structures, differential scanning calorimetry thermogravimetry (DSC-TG) (Setaram, Themys one +) was used to study the response of the synthesized biopolymer to heating, and field-emission SEM (FE-SEM) (QUANTA FEG 205) was used to observe the microstructure the biopolymer.

### Ultrasound-assisted esterification of CMC

CMC was modified by esterification *via* an eco-friendly ultrasound-assisted procedure using three alcohols with different carbon-chain lengths (methanol, butanol and octadecanol). The synthesis of the hydrophobic CMCs was adapted from similar literature methods.^29–32^ Briefly, three conical flasks with caps were prepared. To the first, CMC (1 g) in methanol (200 mL) was added (CMC-C1); to the second, butanol (450 mL) was added (CMC-C4); and to the third, octadecanol (4.5 g) in methanol (200 mL) was added (CMC-C18). After that, concentrated sulfuric acid 96% (1 mL) was added to each one, and then the flasks were placed in an ultrasonic water bath set at 40 °C for 2 h. After this time, the newly synthesised hydrophobic CMC products were filtered, then washed with about 50 mL of ethanol (70%) twice, followed by acetone. The new products were then dried in an oven at 40 °C for 1 h. The physical and chemical properties of the synthesised biopolymers were determined using different characterisation methods (FT-IR, XRD, DSC TG, and SEM).

### Determination of fat% removal

To investigate the performance of the synthesized hydrophobic CMCs in the removal of fat, chicken (2 g, fat sample) was extracted using the same method previously described. Next, the obtained fat was weighed after evaporating the solvent, and was then dissolved again using ethyl acetate before adding the hydrophobic CMCs. The mixture was shaken for 10 min in a rotary shaker, the solvent was evaporated, and then the sample was weighed to determine the remaining fat. The fat% removal was calculated by dividing the weight of the fat after using the hydrophobic CMCs by the initial weight of the fat, then multiplying the attained value by 100. This process was repeated three times and the average percentage was reported for each biopolymer.

### Nitrofuran (NF) metabolites analysis

The extraction of NF metabolites from tissue sample, including the derivatization process, were conducted according to a previous method reported in the literature, with slight modifications.^33^ Briefly, a sample (2 g) of minced and homogenized chicken was weighed in a test tube, then fortified with NF metabolites and isotope internal standards at a level of 100 μg kg^−1^. After that, DI water (4 mL), 1 M HCl (0.5 mL), and 50 mM 2-NP (150 μL) were added sequentially to the sample. The mixture was then vortexed and placed in a dark and closed water bath at 50 °C for 2 h. Next, the pH was adjusted by adding 0.1 M K_2_HPO_4_ (5 mL) and 1 M NaOH (0.4 mL). The sample was then vortexed again, and ethyl acetate (5 mL) was added to extract the target analytes. After shaking for 1 min, the test tube was placed in a centrifuge at 4000 rpm at 4 °C for 5 min. The upper layer of the sample was then collected in a clean test tube and evaporated to dryness using an evaporation system. After that, the analytes were re-dissolved in MeOH (400 μL) and 0.1 g of the hydrophobic CMCs were added. The mixture was then shaken for 10 min, before being centrifuged again at 4000 rpm at 4 °C for 5 min. Finally, the upper layer of the sample was transferred to an LC vial.

### LC-MS/MS analysis

The instrumentation setup was displayed under ideal conditions, the used system was +ESI LC-MS/MS AB Science Triple Quad 6500+ equipped with an Agilent UPLC 1290. The separation of NF compounds was achieved at flow rate of 0.2 mL min^−1^, with injection volume 5 μL. The mobile phase was a gradient containing 80% 0.5 mM ammonium acetate in methanol (eluent A) and pure methanol (eluent B), with gradient programme as follows: 0 min: 20% B, 7 min: 55% B, 8 min: 20% B, 13 min: 20% B. The total run was 13 min including the washing step. The analytical column was reverse phase (Luna 150 × 2 mm, 3 μm C18(2) 100 Å), which was operated at column oven at 40 °C. The mass spectrometry was Triple Q 6500+ with electrospray ionization at positive mode (+ESI). The ion source parameters were as follows: temp. 550 °C, ion spray voltage 5500 V, curtain gas pressure 35 psi, collision gas 8 psi, ion source gas 1 pressure 50 psi, ion source gas 2 pressure 60 psi. The detection of NF analytes was done by Multiple Reaction Monitoring (MRM) mode, all MRMs and compound-dependent parameters were illustrated in Table 1.

**Table 1:** The MRMs parameters for NF metabolites

Analyte	Precursor ion (*m*/*z*)	Product ions (*m*/*z*)	DP (V)	CE (eV)
2-NP-SEM	209.1	166.2^a^, 192.2	46	13, 25
2-NP-SEM-13C,15N_2_	212.2	168.2	46	15
2-NP-AOZ	236.0	134.1^a^, 104.0	46	17, 30
2-NP-AOZ-D4	240.4	134.1	100	17
2-NP-AHD	249.0	134.0^a^, 178.1	46	20, 20
2-NP-AHD-13C3	252.0	134.0	111	15
2-NP-AMOZ	334.8	291.1^a^, 262.1	116	15, 25
2-NP-AMOZ-D4	339.7	296.0	116	17

a Quantifier ion, DP (declustering potential), CE (collision energy).

## Results and discussion

### Synthesis

**Fig. 1:**
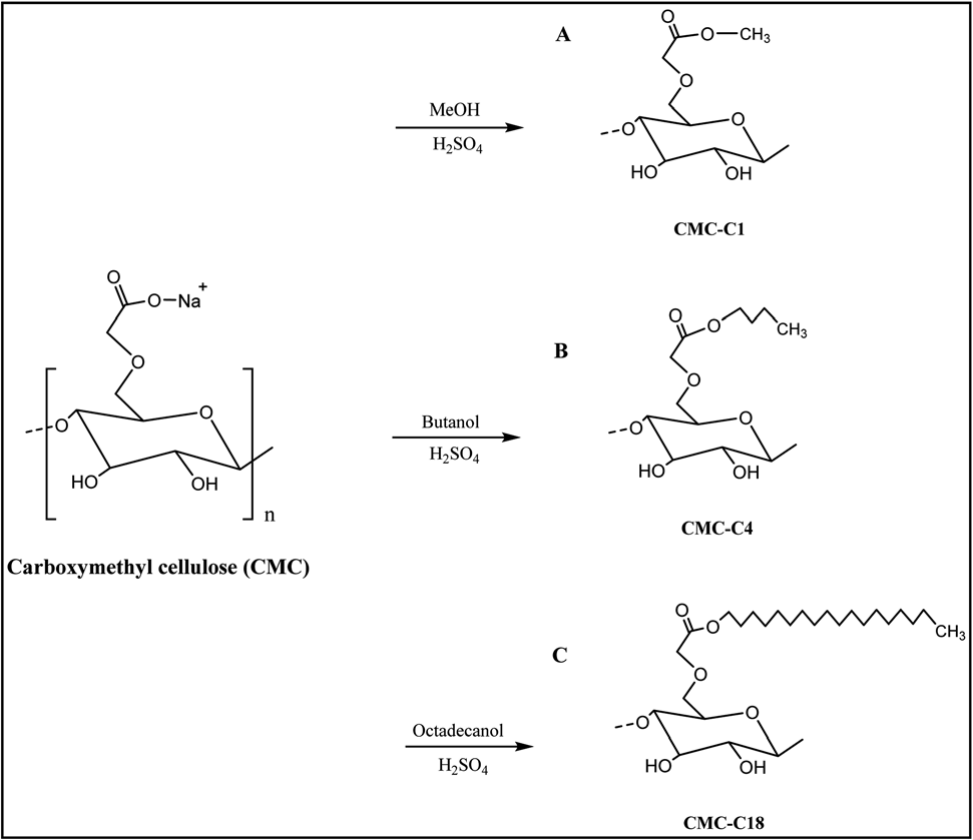
The reaction mechanism of hydrophobic CMCs synthesis, (A) CMC-C1, (B) CMC-C4, (C) CMC-C18.

The synthesis of the hydrophobic CMCs was conducted by esterification of CMC with three different alcohols to endow the biopolymer with hydrophobic properties. First, esterification was performed with methanol, which contains one carbon (CMC-C1). Second, butanol was added to the biopolymer to form CMC linked to a chain containing four hydrocarbons (CMC-C4). Finally, to obtain CMC with a long hydrocarbon chain, octadecanol was added to obtain a biopolymer with 18 hydrocarbons (CMC-C18), which increased the hydrophobicity of CMC. The synthesised hydrophobic CMCs are shown in Fig. 1.

### Characterisation

The structures and physicochemical properties of the synthesized hydrophobic CMCs were verified using several characterization methods.

#### FT-IR spectroscopy analysis

**Fig. 2:**
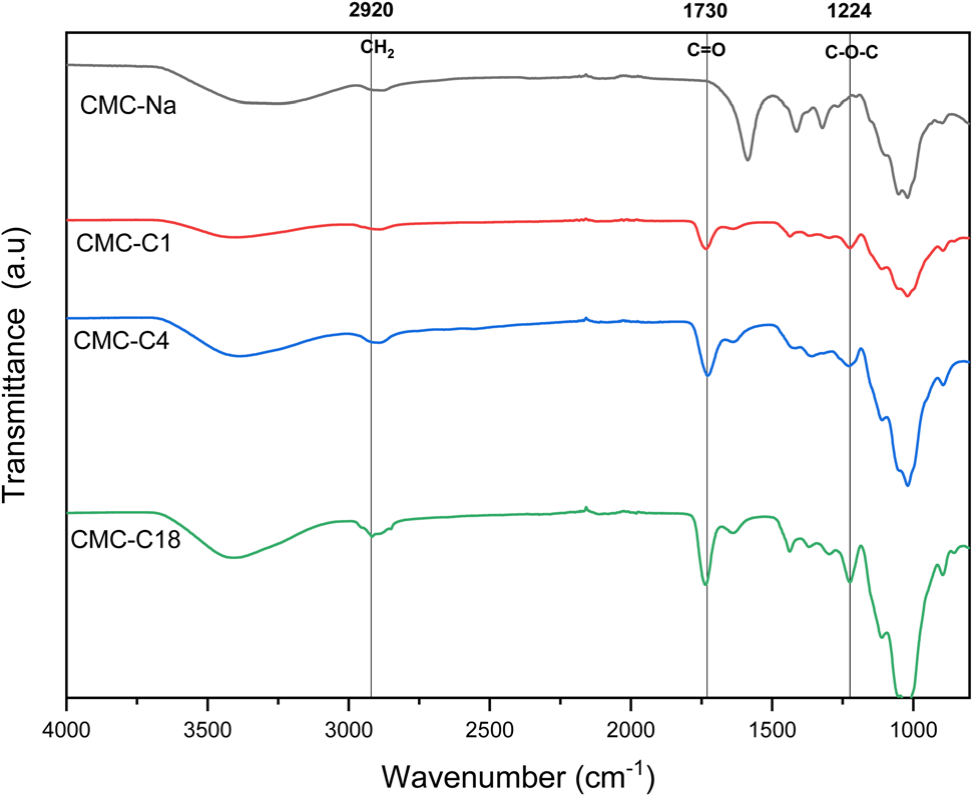
FT-IR spectra of hydrophobic CMCs and pure CMC-Na.

The successful synthesis of the hydrophobic CMC biopolymers was confirmed by FT-IR spectroscopy. Fig. 2 shows the FT-IR spectra of the hydrophobic CMCs alongside that of pure CMC-Na. Two main characteristic bands can be seen clearly from the spectra, confirming the esterification of the CMCs with the different alcohols (methanol, butanol, and octadecanol). First, the main characteristic band can be observed at 1224 cm^−1^, which is related to the stretching of the ester group (C–O–C). This is observed for all the hydrophobic CMCs, but not for pure CMC-Na. Second, the other main characteristic band related to the stretching vibration of the C=O group can be observed at 1730 cm^−1^, which is also observed for pure CMC-Na shifted to 1600 cm^−1^ due to the esterification with the alcohol. Hence, the successful esterification of CMC for all the hydrophobic CMCs was confirmed by the presence of both characteristic bands at 1224 cm^−1^ and 1730 cm^−1^. In addition, additional bands confirm the conserving of the structure of CMC, such as the band at 2920 cm^−1^ related to the C–H stretching of a CH_2_ group, as well as the broad band between 3200 cm^−1^ and 3600 cm^−1^ attributed to the stretching vibration of O–H. All the mentioned characteristic bands are consistent with the bands previously reported for similar structures reported in the literature.^29,34–38^

#### XRD analysis

**Fig. 3:**
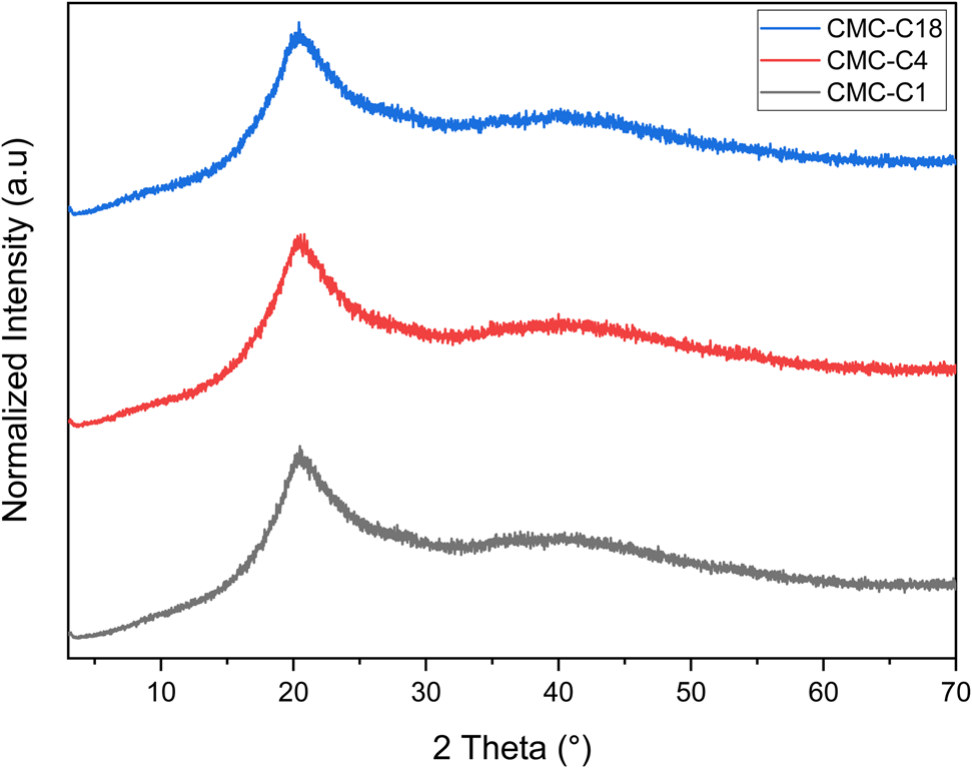
XRD spectra of hydrophobic CMCs.

The crystal structure of the fabricated hydrophobic CMCs was investigated by XRD to understand the crystallinity nature of the synthesized materials whether they are crystalline or amorphous. Fig. 3 shows the XRD patterns of the hydrophobic CMCs, from which it is clear that they exhibit the same diffraction pattern with a main characteristic broad peak at around 2*θ* = 15–30° and a minor broad pattern at 2*θ* = 40°. Similar results have been reported in previous studies for CMC, where it showed broad beak at 21°.^39–43^ The esterification of CMC with various hydrocarbon chains showed another minor broad peak at 40°, which slightly increased the crystallinity of the produced materials. Therefore, the XRD patterns show the fabricated hydrophobic CMCs to be semi-crystalline in nature, indicating their structural stability.

#### TGA measurements

**Fig. 4:**
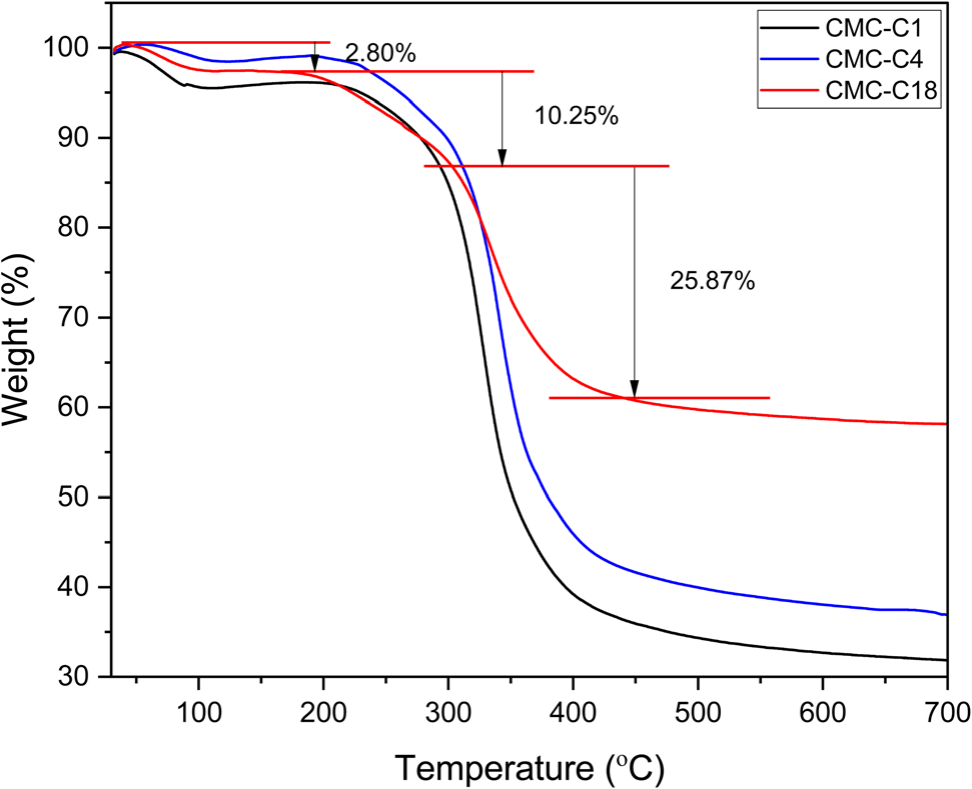
Thermogravimetric analysis (TGA) curves of hydrophobic CMCs.

The thermal stability of the hydrophobic CMCs was investigated using TGA, with the recorded thermograms shown in Fig. 4. The decomposition of the fabricated hydrophobic CMCs was observed to occur in three stages. In the first stage, an initial weight loss of around 2.8% was observed for the hydrophobic CMCs in the range of 80–120 °C, attributed to minor degradation due to the loss of absorbed moisture from the materials.^7^ The second stage occurred when the temperature reached 180 °C, with a gradual loss of around 10% of sample weight due to the loss of inorganic moieties.^44^ In the third stage, high mass degradation occurred at >300 °C due to pyrolysis of the cellulosic backbone.^7^ Different results were observed at this point for the hydrophobic CMCs; CMC-C18 exhibited the least degradation at around 25% weight loss, while CMC-C4 and CMC-C1 exhibited the most degradation at weight losses of around 46% and 54%, respectively. From these results, it can be concluded that CMC-C18 exhibits higher thermal stability than the other hydrophobic CMCs due to its chemical structure featuring a long hydrocarbon chain that supports the polymer skeleton. In addition, it was noted that CMC-C4 also exhibited better thermal properties than CMC-C1 due to its polymer structure.

#### Morphology analysis

**Fig. 5:**
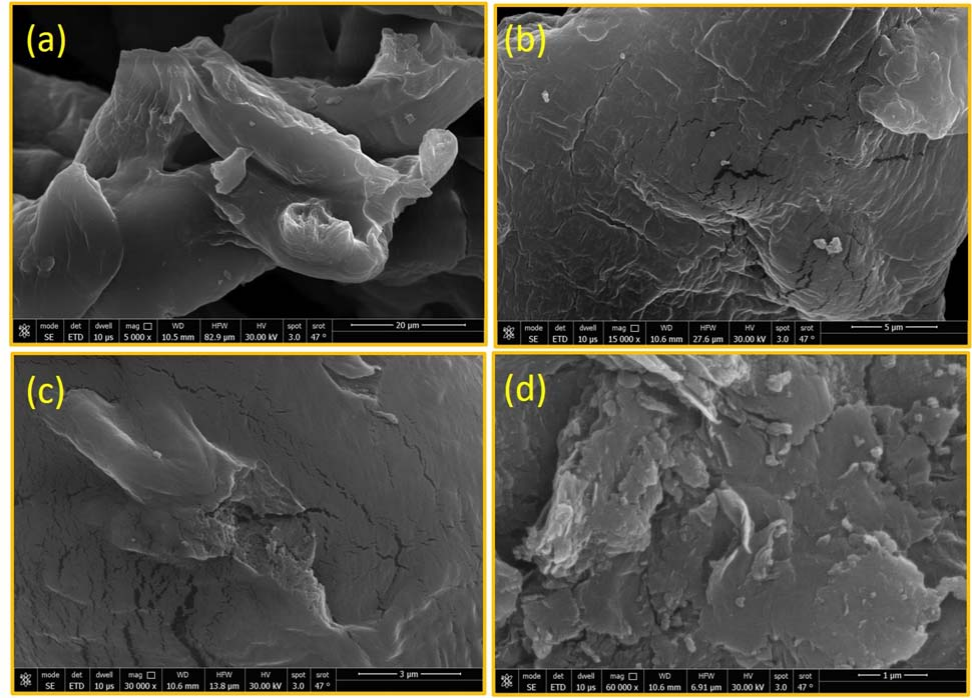
SEM micrographs of CMC-C18 with several magnification at (a) 5000× (b) 150 00× (c) 300 00× (d) 600 00×.

The surface morphology of the hydrophobic CMCs was studied by SEM. Fig. 5 shows the obtained SEM images of CMC-C18 at various magnifications as a representative hydrophobic CMC. In general, the surface morphology of pure CMC is smooth and uniform with tiny ordered cracks present on the polymer surface, as described in many previous studies.^5,45,46^Fig. 5a shows the magnified SEM image of CMC-C18 at 5000×, from which the biopolymer has a smooth and soft appearance. Fig. 5b shows the magnified SEM image of CMC-C18 at 150 00×, where the sample surface has a hard appearance with tiny voids. At a higher magnification of 300 00× (Fig. 5c), the sample surface is mountainous in appearance with deep voids. At the highest magnification of 600 00× (Fig. 5d), the surface of CMC-C18 shows aggregated slices and particles like hills. These changes in the surface of CMC-C18 confirm the successful chemical modification of the biopolymer and its increased surface area, which boost its absorbance and enhance its interaction with the target.

#### NF metabolites application

**Fig. 6:**
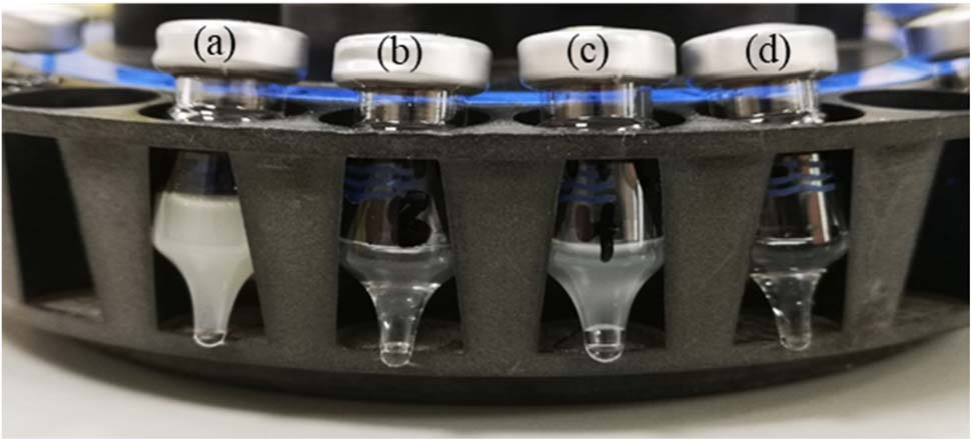
The physical appearance of the final extractor of nitrofuran metabolites determination, (a) QC (without Hydrophobic CMCs), (b) CMC-C18, (c) CMC-C4, (d) CMC-C1.

The hydrophobic CMCs were tested in a real sample analysis application to determine their performance as sample clean-up materials. The chicken samples were spiked with 1 ng kg^−1^ of all of the NF metabolites, which is the MRPL for NF residues. The performance of the hydrophobic CMCs was determined first by the physical appearance of the final extraction. Fig. 6 shows the physical appearance of the final extraction, where (a) is the quality control (without hydrophobic properties), (b) is CMC-C18, (c) is CMC-C4, and (d) is CMC-C1. These images clearly indicate the positive effect of using the hydrophobic CMCs as sample clean-up materials. The turbidity of the quality control sample was clearly observed, while the hydrophobic CMCs samples were clearer and purer, as shown in Fig. 6. Further, among the hydrophobic CMCs, CMC-C18 and CMC-C1 showed clearer final solutions than CMC-C4. In general, the hydrophobic CMCs removed fats from the tested sample and reduced the undesirable effects of the sample matrix.

**Fig. 7:**
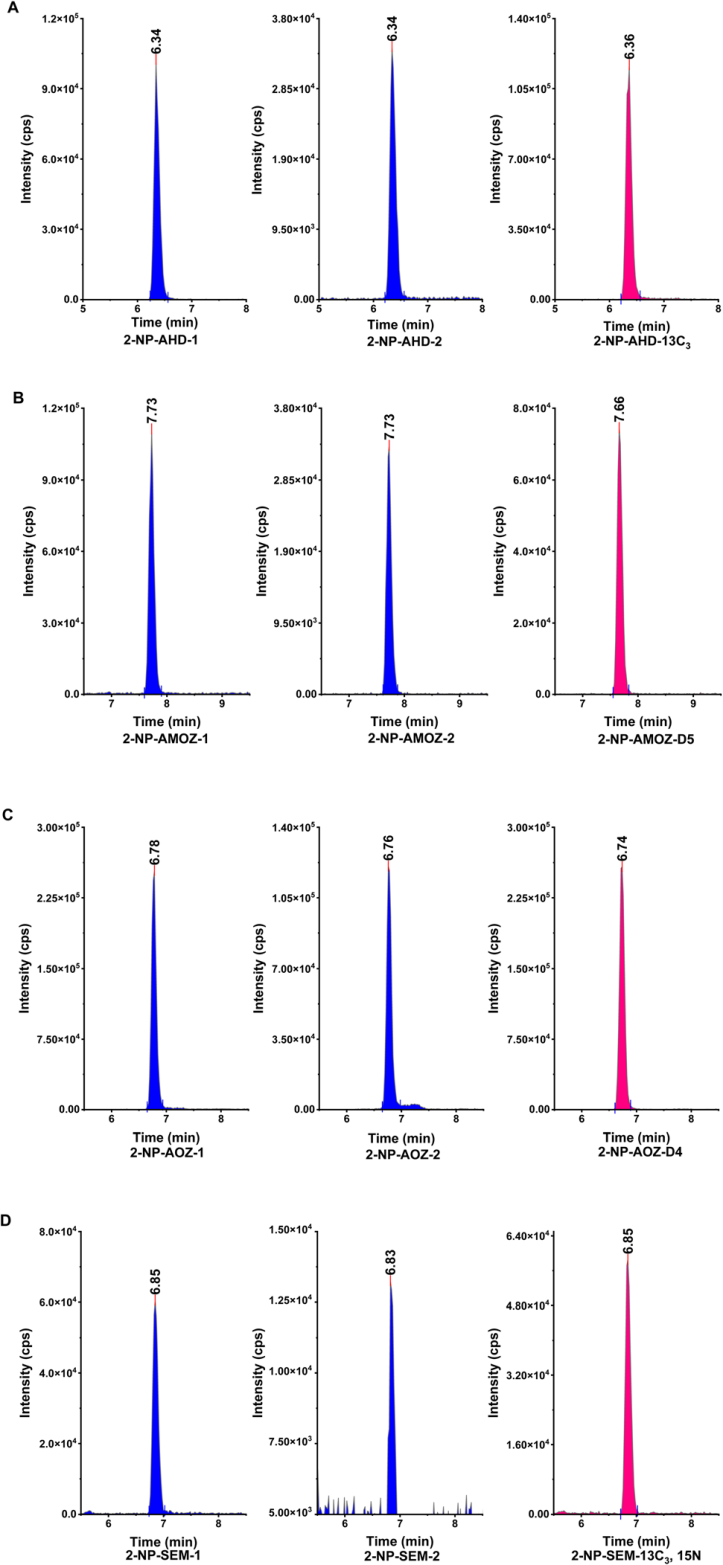
The chromatograms of nitrofuran metabolites MRMs and isotope internal standard, (A) 2-NP-AHD, (B) 2-NP-AMOZ, (C) 2-NP-AOZ, (D) 2-NP-SEM.

The second investigation in the performance of the hydrophobic CMCs was undertaken by determining the effect of the hydrophobic CMCs on the target analytes. Tissue sample was spiked with the NF metabolites (SEM, AOZ, AMOZ, and AHD) and then the recoveries were studied after applying the hydrophobic CMCs as a clean-up step. Table 1 illustrates the concentrations and recoveries of NF metabolites using the hydrophobic CMCs. The results show that the NF metabolite concentrations and recoveries are between 0.97 and 1.17 μg kg^−1^ and 97–117%, respectively, excluding the recovery of AMOZ in CMC-C4, which was around 157%. This result could be an overestimation due to the used sample interference. Overall, the hydrophobic CMCs were able to remove the unwanted matrix, especially fats, and maintain the target analytes without effecting their extraction. All recoveries were acceptable and met the specifications stipulated in EU regulation 2002/657/EC,^47^ which states that recovery should be between 50 and 120% for concentrations of ≤1 μg kg^−1^. The multiple reaction monitoring (MRM) chromatograms of NF metabolites and their isotope internal standards in a spiked sample are shown in Fig. 7.

It is important to note that the regeneration and reusability of hydrophobic CMCs are not preferred due to they were interacted with fat matrix of food sample and will be precipitated and discard at the end of analysis. The rewash or removing of the fat content by organic solvent could damage the structure of hydrophobic CMCs or remove it as well with fat molecules.

#### Fat% removal application

Calculating the percentage of fat removed from tissue samples was another application used to investigate the performance of the hydrophobic CMCs. Table 2 shows the results of the percentage of fat removal using the hydrophobic CMCs. It can be seen observed that the hydrophobic CMCs removed a high quantity of fat from the samples, in the range of 88–94%. The highest fat removal percentage was observed for CMC-C18 at 94.2%, due to the length of its hydrocarbon chain meaning that it interacts easily with fatty acid chains. In this case, the hydrophobic interaction was stronger than for the other hydrophobic CMCs. However, although CMC-C4 and CMC-C1 have shorter hydrocarbon chains, they also showed good performances, removing 91.1 and 88.5% fat from the tested samples, respectively.

**Table 2:** The recoveries study of NF metabolites using hydrophobic CMCs

Analyte	Spike level (μg kg^−1^)	CMC-C1			CMC-C4			CMC-C18		
		Conc. (μg kg^−1^)	Recovery (%)	CV (%)	Conc. (μg kg^−1^)	Recovery (%)	CV (%)	Conc. (μg kg^−1^)	Recovery (%)	CV^a^ (%)
AOZ	1	1.12	112	6.7	1.02	102	3.9	1.17	117	2.8
AMOZ		1.05	105	5.3	1.57	157	11.9	1.15	115	4.0
AHD		1.08	108	6.3	0.97	97	4.3	1.04	104	6.7
SEM		1.03	103	5.3	1.007	100	4.2	1.04	104	5.9

a CV: coefficient of variation.

**Fig. 8:**
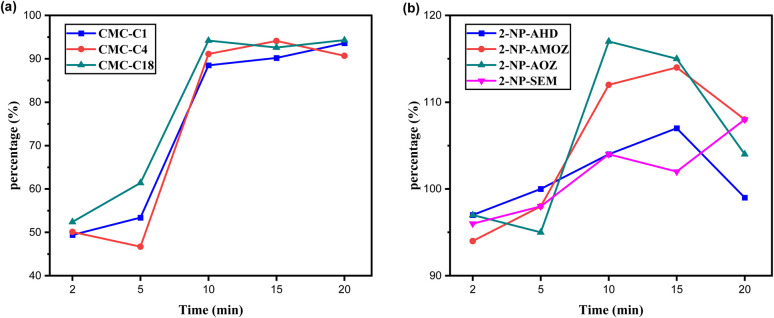
Effect of shaking time on, (a) fat% removal, (b) nitrofuran metabolites recovery.

The shaking time after adding the hydrophobic CMCs was studied and optimised due to the significant effect it has on fat removal and maintaining the target analytes. Shaking times of 2, 5, 10, 15 and 20 min were selected for both applications. Fig. 8a shows the fat% removal over three experiments. It can be seen that the hydrophobic CMCs removed 45–65% of fat content after 2 to 5 min of shaking, which increased to 85–95% by 10 min. After 15 to 20 min of shaking, the fat% removal slightly increased for CMC-C1 and CMC-C4 and slightly decreased for CMC-C18. Therefore, 10 min of shaking was determined to be the optimal time needed to remove fat from the sample.

Regarding the NF metabolite application, the same shaking times were studied for types of the hydrophobic CMCs to evaluate their performance. All the shaking times were able to maintain NF recoveries of 94–117%, as shown in Fig. 8b. So, the optimal shaking time of 10 min for fat% removal was determined to be suitable for NF recovery Table 3.

**Table 3:** The fat% removal of Hydrophobic CMCs

Hydrophobic CMCs	Wt. fat (mg)		Removal (%)	CV^a^ (%)
	Before	After		
CMC-C1	5.55	0.64	88.5	1.7
CMC-C4	5.93	0.53	91.1	0.7
CMC-C18	11.45	0.66	94.2	0.8

a CV: coefficient of variation.

#### Method validation

After the method optimization, the NF metabolites analysis method was validated using CMC-C18 that provided the best obtained results. The method validation was conducted in compliance with EU regulation 2002/657/EC.^47^ The validation was done for three level; 0.5, 1 and 1.5 μg kg^−1^ to cover the interesting levels around MRPL. Table 4 shows the results of the main performance characteristics of method validation. The selectivity and specificity were successfully evaluated by spiking the matrix with low level. There is no interference that can affect on the peak retention time and can be identified easily. The LOD and LOQ provide satisfactory results that met the requirement according to Codex,^48^ which come below 0.2 and 0.4 μg kg^−1^ for LOD and LOQ respectively. The trueness was determined by the recovery percentage for each NF metabolites, they were ranged between 83.3 to 104.3% that complies with regulation as well. For precision, intra-day precision (repeatability) was applied by coefficient of variation (CV%) for NF metabolites, the obtained results were lower that 10%, ranged between 3.0 to 6.5%. They showed high precision for this method for the three levels. Finally, the linearity was assessed by correlation coefficient (*r*^2^), all NF metabolites showed *r*^2^ higher than 0.99.

**Table 4:** The results of performance characteristics of the method validation

Analyte	Spiking level	Recovery	Intra-day precision	Correlation coefficient	LOD	LOQ
	(μg kg^−1^)	%	(% CV)	(*r*^2^)	(μg kg^−1^)	(μg kg^−1^)
AOZ	1.5	103.3	4.8	0.9957	0.09	0.28
	1	102.9	5.5			
	0.5	93.8	5.9			
AMOZ	1.5	98.7	6.4	0.9985	0.06	0.20
	1	99.6	5.5			
	0.5	96.2	4.2			
AHD	1.5	99.3	4.5	0.9966	0.08	0.27
	1	104.3	3.1			
	0.5	89.5	6.0			
SEM	1.5	99.9	4.7	0.9944	0.08	0.27
	1	95.7	4.9			
	0.5	83.3	6.5			

Compared to others sorbents or processes of fat removal such as freezing, SPE and LLE, hydrophobic CMCs materials are green based on biopolymer. They are also reducing the analysis time, low cost and small quantity to remove fats. Table 5 summaries the most common sorbents or processes that have been used in fat removal of food sample analysis.

**Table 5:** The comparison of common clean-up methods used in fat removal

No.	Sorbent/process	Technique	Application	Removal%	Adv.	Dis-adv.	Ref.
1	Freezing-lipid filtration	Freezing	Endocrine-disrupting phenols in fish sample	90	Low-cost, simple	Time-consuming, interference with analyte	27 and 28
2	Hexane	LLE	Pesticide analysis in high-fat samples	47	Fast, simple	Large amount of solvents, low efficiency	24
3	Acidic or basic treatments	LLE	Steroids in animal tissues	NA	Simple	Large amount of solvents, low efficiency, many interference	25 and 26
4	C18, silica, phenyl	Automatic TurborFlow online SPE column	Veterinary drug residues in milk	NA	Reducing-error source, high efficiency	High-cost, instrumentation, large amounts of solvent, time-consuming, contamination	49
5	PSA, C18, MgSO4	d-SPE	Sulfonamides in bovine liver	NA	Simple, fast, kit package	High cost, analytes suppression	50
6	EMR-lipid	d-SPE	Mycotoxins, acrylamide and 5-hydroxymethylfurfural in biscuit	NA	Simple, fast, kit package, nanomaterial	Unknown material, high cost	20 and 21
7	Florisil	SPE column	Pesticide residues in olives and olive oil	NA	Simple, commercially available	Time consuming, large amount of solvents	22
8	Hydrophobic CMCs biopolymer	d-SPE	Nitrofuran metabolites in fat sample food	88.5–94.2	Simple, fast, green	Need synthesis	This work

## Conclusion

In this work, CMC was chemically modified by esterifying it with methanol, butanol, and octadecanol in the presence of sulfuric acid to obtain the respective hydrophobic biopolymers. The synthesis occurred successfully, then confirmed by characterization techniques. The performance of the synthesised hydrophobic CMCs was investigated by measuring the fat removal percentage and their use as clean-up sorbents in NF metabolite determination. The hydrophobic CMCs showed high performance by removing fat from food sample extracts. Detection of NF metabolites were investigated using the hydrophobic CMCs, and the method was validated using CMC-C18 as clean-up sorbent. The use of the hydrophobic CMCs as sorbents in food analysis applications, especially for foods with a high fat content, shows promise as a fast, low-cost, eco-friendly, and simple method. It is thus highly recommended to use hydrophobic CMCs in other food analysis applications, either as a removal sorbent or to extract target analytes. Moreover, the hydrophobic CMCs could be further developed using nanocomposites to produce nanomaterials with specific and distinct properties.

## Disclaimer

“The views expressed in this paper are those of the author(s) and do not necessarily reflect those of the SFDA or its stakeholders. Guaranteeing the accuracy and the validity of the data is solely the responsibility of the research team”.

## Author contributions

O. A. Thabet: methodology, investigation, writing – original draft; F. S. Al Muzini and A. M. Atiya: methodology, investigation; K. A. Alamry and M. A. Hussein: conceptualization, investigation, writing – review & editing; R. Hoogenboom: review & editing – final draft.

## Conflicts of interest

There are no conflicts to declare.

## Supplementary Material
